# Glaucoma Screening in the Haitian Afro-Caribbean Population of South Florida

**DOI:** 10.1371/journal.pone.0115942

**Published:** 2014-12-30

**Authors:** Christine L. Bokman, Louis R. Pasquale, Richard K. Parrish, Richard K. Lee

**Affiliations:** 1 Bascom Palmer Eye Institute, University of Miami Miller School of Medicine, Miami, FL, United States of America; 2 Massachusetts Eye and Ear Infirmary, Harvard Medical School, Boston, MA, United States of America; 3 Channing Division of Network Medicine, Brigham and Women’s Hospital, Harvard Medical School, Boston, MA, United States of America; Casey Eye Institute, United States of America

## Abstract

**Objective:**

To evaluate the presence of clinical signs consistent with suspected glaucoma in Haitian Afro-Caribbean individuals residing in South Florida who do not receive regular eye examinations.

**Design:**

Retrospective, cross-sectional study.

**Methods:**

SETTING: Community health center in the Little Haiti district of Miami, Florida. PATIENT POPULATION: We reviewed medical records and screening forms from five health screenings between October 2011 to October 2013 of 939 Afro-Caribbean individuals older than 18 years, who were never diagnosed with glaucoma or had an eye examination within the last ten years. PROCEDURES: Measurements of distance visual acuity (VA), intraocular eye pressure (IOP), central corneal thickness (CCT), cup-to-disc ratio (CDR), frequency doubling technology (FDT) perimeter visual field (VF).

**Main Outcome Measures:**

Proportion of glaucoma suspects, based on IOP greater than or equal to 24 mm Hg or CDR greater than or equal to 0.7 in either eye, and determinants of CDR and IOP.

**Results:**

One hundred ninety-one (25.5%) of 750 patients were identified as glaucoma suspects. Glaucoma suspects were common in both the youngest and oldest age groups (<40 years, 20.9%; 95% confidence interval [CI], 17.9–23.9; >70 years, 25.0%; 95% CI, 21.8–28.2) and higher in men than women less than 70 years; the reverse was true after 70 years. Among all patients, mean IOP was 19.2±4.5 mmHg, mean CDR was 0.37±0.17, and mean CCT was 532±37.1 µm. In multiple linear stepwise regression analysis, determinates of increased CDR included increasing age (*P* = 0.004), lack of insurance (*P* = 0.019), and higher IOP (*P*<0.001), while increasing CDR (*P*<0.001) and thicker CCT (*P*<0.001) were associated with higher IOP.

**Conclusions:**

This first glaucoma survey in a U.S. Haitian Afro-Caribbean population indicates glaucoma suspect status is high across all age groups, and suggests glaucoma monitoring in people less than 40 years of age is indicated in this population.

## Introduction

Glaucoma is the leading cause of irreversible blindness worldwide [1]. The most common subtype, primary open-angle glaucoma, is characterized by slowly progressive optic nerve atrophy that can ultimately lead to blindness [2]. Estimates show more than 2.5 million people in the United States suffer from glaucoma [3]. Among ethnic groups within the U.S., large differences exist in glaucoma prevalence. Several studies have shown that glaucoma more frequently affects African Americans [4]–[6] and Latinos [7]. No study to date has investigated the severity of glaucoma among Afro-Caribbean people residing in the U.S., a population distinct from African Americans. Afro-Caribbeans living outside the U.S. suffer from a high rate of glaucoma [8], [9], and this population is affected at earlier ages [8]–[11]. Among Afro-Caribbeans living in the U.S., a population that has nearly tripled over the last twenty years [12], it is unknown if this population has comparable morbidity to Afro-Caribbeans outside the U.S.

Since glaucoma is largely asymptomatic until the disease is advanced, screening represents a logical and clinically important approach to identify early stage cases and reduce visual disability and legal blindness. Recently, the United States Preventative Task Force (USPTF) updated screening recommendations for glaucoma [13] and found insufficient evidence to support screening [14]. By narrowing the focus of screening on a high-risk population, Ladapo and his coworkers investigated the hypothetical effect of screening on visual outcomes in African Americans and found only a modest impact on visual impairment and blindness [15]. These conclusions may not necessarily apply to the Afro-Caribbean population because the severity of disease remains unknown. Screening would be especially important if glaucoma affects this population at younger ages.

To address this knowledge gap, data from a community outreach effort were analyzed to determine the extent of suspected glaucoma in an Afro-Caribbean community living in South Florida – the Haitian Afro-Caribbean population. The patients included in the study regularly seek medical care at the Center for Haitian Studies, a local community health center, but have never received an eye exam within the last ten years nor the diagnosis of or treatment for glaucoma. The aim of this study is two-fold: The first is to describe the extent of suspected glaucoma in an at-risk population in the U.S. The second is to investigate the assumption that the reason why people of African decent suffer from higher rates of glaucoma-associated blindness is because the disease occurs earlier in life – no study has included participants less than 40 years old to test this hypothesis.

## Methods

### Study Population

This retrospective study was approved by the Institutional Review Board of the University of Miami Miller School of Medicine (ID# 20130573), and conducted in accordance with the Declaration of Helsinki. We reviewed medical charts and screening forms of patients who attended glaucoma screenings that took place at a community health center in the Little Haiti District of Miami, Florida, from October 2011 through October 2013. This center provides general healthcare, including obstetrics/gynecology and pediatrics, but eye care is not provided. Screening forms were provided by the Friends of The Congressional Glaucoma Caucus Foundation. Glaucoma screenings were a part of three general health and two glaucoma screenings, which were advertised throughout the local community three weeks in advance via public radio, the distribution of flyers, and newspaper advertisements. All people who attended the general health screenings voluntarily chose to participate in glaucoma screening. All participants were given written, informed consent for their clinical records to be used in this study, to which all consented.

A total of 939 patients participated in the screenings. The average number of patients per screening was 188±38. A total of 496 women (55.5%) and 398 men (44.5%) participated. Patients who indicated they had glaucoma or were a glaucoma suspect, received glaucoma treatment in the past, or received an eye exam within the last ten years were excluded from this analysis. Patients 18 years or younger were also excluded from analysis. All subjects identified themselves as Haitian, black, or Caribbean.

### Examination Procedures

Measurements of visual acuity (VA), intraocular eye pressure (IOP), central corneal thickness (CCT), vertical cup-to-disc ratio (CDR) and visual field (VF) were performed. Distance VA was assessed with an individual’s habitual refraction in each eye using Snellen eye charts that were corrected for 10 feet. If patients did not have glasses or visual acuity was worse than 20/40 with or without glasses, a pinhole visual acuity was tested. Intraocular pressure was measured with a Tonopen (Reichert Technologies, Depew, NY) and CCT was measured by ultrasound pachymeter (Pachette 3, DGH Technology, Exton, PA). Vertical cup-to-disc ratio was estimated via direct ophthalmoscopy on non-dilated eyes. Optic nerves were also photographed with a non-mydriatic fundus and optic nerve head camera at two of the glaucoma screenings when the camera was available (372 [49.6%] of 750 patients). A designated ophthalmologist and ophthalmology resident read all optic nerve head (ONH) and fundus photographs. Cup-to-disc ratios were comparable between those observed by direct ophthalmoscopy on non-dilated eyes and independently read by ONH photographs by an independent ophthalmologist and/or ophthalmology resident at a site distant from the vision screening (Pearson’s coefficient = 0.93 [*P*<0.001] for right eyes and 0.89 [*P*<0.001] for left eyes). Visual field was measured using a Frequency Doubling Technology (FDT) perimeter (Humphrey FDT, Welch Allyn, Skaneateles, NY) in C-20-5 screening mode. The number of sectors assigned a probability of <5% or worse on the Total Deviation plots were used to classify VF results as follows: “normal” for 0 or 1 sector; “suspect” for 2 sectors; and “abnormal” for 3 or more sectors. After both eyes were tested to assess VF, the test was repeated if results were unreliable as indicated by the number of false positives greater than 1 out of 3 or if 50% of sectors were abnormal. Residents, glaucoma and neuro-ophthalmology fellows, trained medical students and ophthalmologists from the University of Miami Miller School of Medicine performed these measurements.

We extracted non-ophthalmic information during all screenings. Insurance status was self-reported as having insurance or not. Diabetes mellitus status was also self-reported and confirmed with a fasting glucose of greater than or equal to 126 mg/dL for untreated patients from the patient’s medical chart. If using medication for diabetes mellitus, information on self-reported duration of medication use was also collected and confirmed with their medical charts. Hypertension status was self-reported and confirmed using a brachial blood pressure of greater than 140/90 for untreated patients from the patient’s chart. Medication use for hypertension was also confirmed with their medical charts. If patients did not indicate a past or present history of diabetes or hypertension, charts were reviewed to determine if a patient had previous diagnoses of diabetes or hypertension. Patient medical charts included measurements and diagnoses from previous health screenings that did not include glaucoma screening. For new patients, the status of diabetes and hypertension could not be confirmed and were reported as unconfirmed diagnoses of diabetes or hypertension when self-reported.

### Glaucoma Suspect Classification

Patients were defined as glaucoma suspects based on measurements of IOP and CDR. Glaucoma suspects were defined as those with either an IOP greater than or equal to 24 mm Hg or a vertical CDR greater than or equal to 0.7 or glaucomatous changes of the optic disc (i.e. rim thinning, nerve fiber defect) in at least one eye. Those patients who met criteria were classified as glaucoma suspects. A high proportion of patients had unreliable VF test results (127 [17.0%] of 750 patients), so VF data was not used as a criterion. CCT and VA were also included as components of the examination, but were not used to define glaucoma suspects. We did not confirm cases of presumed glaucoma, nor were we able to assess visual impairment or blindness secondary to glaucoma. Patients documented as glaucoma suspects were given referrals with their test results to follow-up with their primary care doctors or to an ophthalmologist, or if they did not have one or lacked health insurance, were given written referrals to Jackson Memorial Hospital for follow-up care.

### Data Analysis

All patient data were entered into a central database and the diagnosis of glaucoma suspect was dichotomized into binary variables. The percentage of glaucoma suspects was calculated as the number of suspects divided by the eligible population. Glaucoma suspects by age and sex were calculated as percentages with 95% confidence intervals. Descriptive data were presented as means with standard deviations because the data set was normally distributed. Differences in patient characteristics, VA, IOP, CDR, and VF between glaucoma suspect cases and non-cases were compared using unpaired student’s t-test for continuous variables and Χ^2^ for categorical variables. Visual acuity results were converted into log_10 _minimum angle resolvable (logMAR) values. Multivariate linear regression models were constructed for identification of independent predictors of CDR and IOP. Determinants were modeled using a linear regression model constructed using a backwards, stepwise technique. Both CDR and IOP determinants were modeled using a linear regression model with input variables of age, sex, insurance status, diabetes, hypertension, CCT, and IOP or CDR. Statistical analyses were performed using IBM SPSS Statistics, version 21 (IBM Corporation; Armonk, NY). All p-values were 2-tailed and P<0.05 was considered statistically significant.

## Results

Of the 939 patients originally screened, 750 (79.9%) patients were included in the study. Excluded patients receive an eye exam within the last ten years (n = 97), received either the diagnosis of glaucoma or glaucoma suspect prior to the vision screening (n = 79), were receiving treatment for glaucoma at the time of the screening (n = 4), or were less than 18 years of age (n = 9). Among the 750 patients included in the study, a glaucoma suspect diagnosis was identified in 191 patients (25.5%). Table 1 presents the characteristics of participants. The mean age was 51 years (range, 19 to 100 years), 336 were men (44.8%), and 146 (19.5%) had a family history of glaucoma or glaucoma suspect in a first- or second-degree relative. Among all patients, the mean IOP was 19.2±4.5 mm Hg, and mean CDR was 0.37±0.17 with a mean CCT of 532±37 µm. Mean logMAR VA was 0.2±0.16, corresponding to a Snellen equivalent of 20/31. In the better-seeing eye, VA was worse than 20/40 in 191 individuals (25.5%, CI 22.2%–28.5%) and equal to or worse than 20/200 in 13 individuals (1.7%, CI 0.8%–2.7%). Abnormal VF results were identified in 284 patients (37.9%, CI 34.4%–41.3%); however, 127 patients (17.0%) had an unreliable test result.

**Table 1:** Baseline Characteristics of Eligible Participants (n = 750).

Age (years), mean, median (range)	51.2, 53.0 (19–100)
Sex (Male)	44.8%
Family history of glaucoma^a^	19.5%
Hypertension	37.6%
Diabetes mellitus	14.5%
IOP (mmHg), mean±SD (median)	19.2±4.5 (19)
≥22 mmHg (95% Confidence Interval)	31.6% (28.3%–34.9%)
CDR, mean±SD (median)	0.37±0.17 (0.3)
≥0.8 (95% Confidence Interval)	4.8% (2.8%–5.7%)
CCT (µm), mean±SD (median)	532.4±37.1 (532)
Visual Acuity (logMAR), mean±SD	0.2±0.16
<20/40 (CI)^b^ [better than]	25.5% (22.2%–28.5%)
≥20/200 (CI)^b^ [equal to or worse than]	1.7% (0.8%–2.7%)
Visual Field, % Abnormal^c^ (95% Confidence Interval)	37.9% (34.4%–41.3%)

IOP = intraocular pressure; SD = standard deviation; CDR = cup-to-disc radio; CCT = central corneal thickness.

a Family history of glaucoma or glaucoma suspect in a first- or second-degree relative.

b Visual acuity in the better-seeing eye.

c Abnormal visual field is 3 or more sector misses.

**Figure 1:**
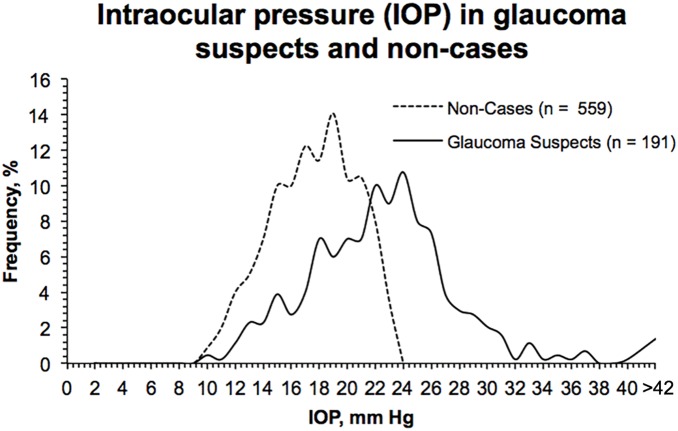
Frequency distribution curves for intraocular pressure in non-cases and glaucoma suspects.

**Figure 2:**
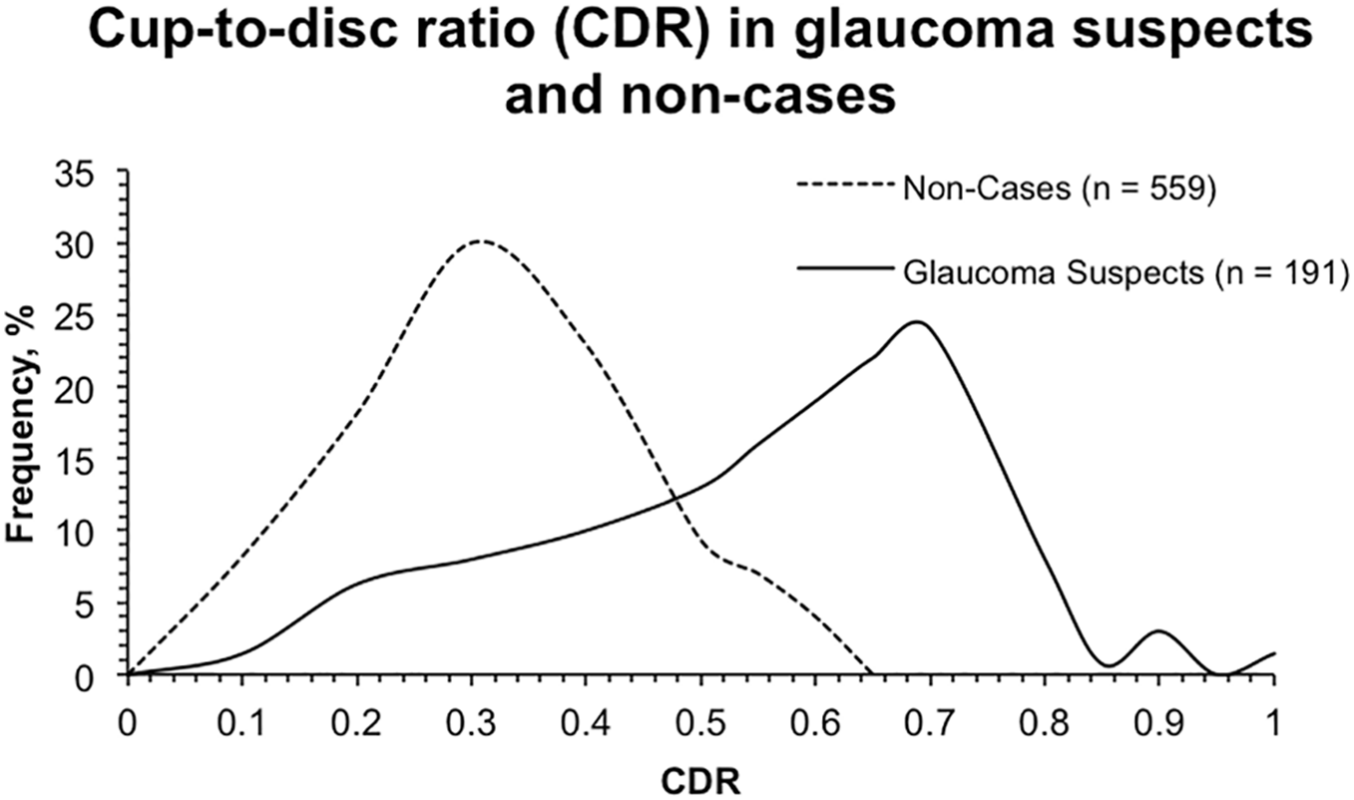
Frequency distribution curves for cup-to-disc ratio in non-cases and glaucoma suspects.

**Figure 3:**
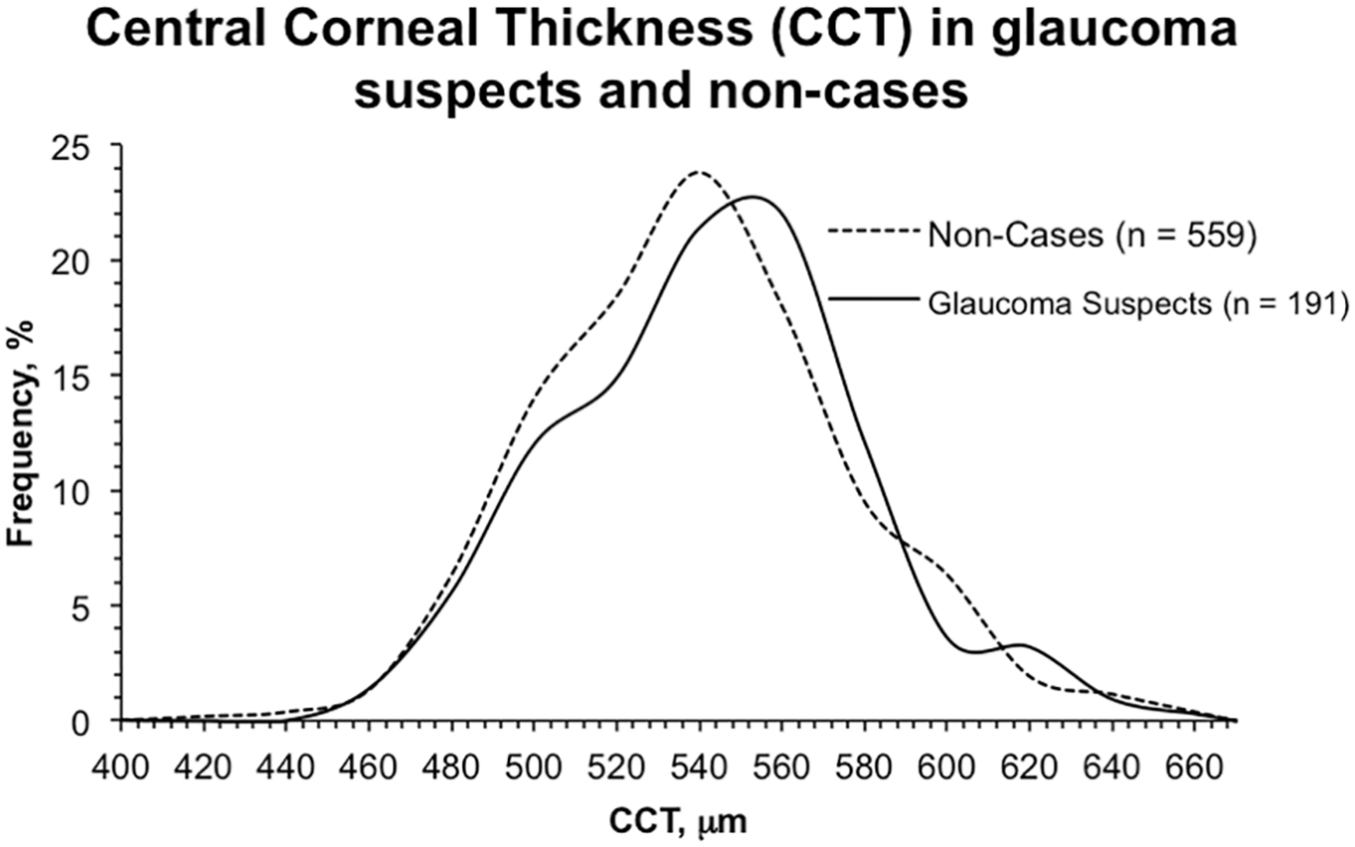
Frequency distribution curves for central corneal thickness in non-cases and glaucoma suspects.

The distributions of IOP, CDR, and CCT among glaucoma suspect cases and non-cases from the worse eye are presented in Figs. 1–3. The mean IOP among glaucoma suspects was 23.6±5.7 mm Hg, compared to 17.1±2.7 mm Hg in non-cases (*P*<0.001). The mean CDR among glaucoma suspects was 0.61±0.20 and 0.27±0.09 (*P*<0.001) among non-cases. The distribution of CDR in glaucoma suspects is wider than the distribution of IOP in glaucoma suspects, which demonstrates that participants were categorized as glaucoma suspects more frequently based on meeting elevated IOP criteria than CDR criteria. Fig. 1 also shows that both non-cases and glaucoma suspects had elevated IOPs. Fig. 3 demonstrates comparable curves for both glaucoma suspects and non-cases based on CCT. Mean CCT among glaucoma suspects was 534±35 µm, compared to 532±32 µm (*P* = 0.42) in non-cases. Mean CCT in participants who only met IOP criteria was 534±31 µm. Mean CCT in patients with IOP greater than 30 mm Hg was 548±12 µm.

Table 2 presents examination findings in glaucoma suspects of VA, CCT, IOP, CDR, and VF. Among 191 patients diagnosed as glaucoma suspects, 46 patients (24.1%) had a CDR greater than or equal to 0.8 in either eye, and 37 patients (19.4%) had an IOP greater than or equal to 30 mm Hg in either eye. Although the criteria for diagnosing patients as glaucoma suspects were based on CDR and IOP, the mean CDR of cases who met the IOP criteria for glaucoma suspect diagnosis (IOP≥24) was 0.55±0.11. The mean IOP of patients who met the CDR criteria for glaucoma suspects (CDR≥0.7) was 22.4±2.5 mm Hg. Among the entire eligible population of 750 individuals, 126 patients (16.8%) met IOP criteria, 106 patients (14.1%) met CDR criteria, and 41 patients (5.5%) met both IOP and CDR criteria for a glaucoma suspect diagnosis. The mean logMAR VA for glaucoma suspect cases was 0.27±0.03 (Snellen equivalent, 20/37), which was worse than the mean logMAR VA of the non-cases group, 0.17±0.01 (Snellen equivalent, 20/30, *P* = 0.004). Ninety-two of 191 glaucoma suspects had VF abnormal results (48.2%, CI 41.5%–54.9%), and 160 of 531 individuals in the non-cases group had abnormal results (30.1%, CI 26.1%–34.2%, *P* = 0.0002).

**Table 2:** Examination Findings in Glaucoma Suspect Cases (n = 191).

Visual Acuity	
Mean, logMAR (±SD)	0.27±0.03
Mean, Snellen Equivalent	20/37
CCT, mean with IOP>30 mmHg (±SD), µm	548±12
IOP	
IOP≥30 mmHg (95% CI), %	19.4 (15.4–23.4)
Mean IOP of cases with CDR≥0.7 (±SD), mmHg	22.4±2.5
CDR	
CDR≥0.8 (95% CI), %	24.1 (20.9–27.3)
Mean CDR of cases with IOP≥24 (±SD)	0.55±0.11
Visual Field, % Abnormal^a^ (95% CI)	48.2 (41.5–54.9)

SD = standard deviation; CCT = central corneal thickness; IOP = intraocular pressure; CDR = cup-to-disc ratio.

a Abnormal visual field is 3 or more sector misses.

Age-specific and gender-specific estimates of suspected glaucoma are presented in Table 3. Suspected disease was elevated among all age groups. Twenty-four of 115 individuals younger than 40 years old were identified as glaucoma suspects (20.9%, CI 17.9%–23.9%), comparable to the 41 of 145 individuals ages 60 to 69 years identified as glaucoma suspects (28.3%, CI 24.9%–31.7%). Suspected disease was higher among men than women in age groups less than 40 years, 40 to 49 years, 50 to 59 years, and 60 to 69 years. Suspected disease was higher in women than men in the 70 years or older group; however, differences were not statistically significant across all age groups (*P*≥0.23). This bivariate analysis represents a composite of CDR and IOP. Therefore, each of these variables was analyzed separately in order to identify determinants of CDR and IOP including risk-adjusted effects of age and sex on CDR and IOP.

**Table 3:** Age and Sex-Specific Rates of Glaucoma Suspect (n = 191).

	Female		Male		Total	
Age Group (years)	No./No. at Risk	GS proportion, % (95% CI)	No./No.at Risk	GS proportion, % (95% CI)	No./No. at Risk	GS proportion, % (95% CI)
<40	16/78	20.5 (17.6–23.4)	8/37	21.6 (24.6–18.6)	24/115	20.9 (17.9–23.9)
40–49	21/95	22.1 (19.0–25.2)	19/73	26.0 (22.8–29.2)	40/168	23.8 (20.6–27.0)
50–59	39/150	26.0 (22.8–29.2)	39/140	27.9 (24.5–31.3)	78/290	26.9 (23.6–30.2)
60–69	20/71	28.2 (24.8–31.6)	21/74	28.4 (25.0–31.8)	41/145	28.3 (24.9–31.7)
≥70	6/20	30.0 (26.6–33.4)	2/12	16.7 (14.0–19.4)	8/32	25.0 (21.8–28.2)
Total	102/414	24.6 (21.4–27.8)	89/336	26.5 (23.2–29.8)	191/750	25.5 (22.2–28.8)

GS = glaucoma suspect.

The determinants of CDR and IOP after adjusting for covariates are presented in Table 4. Models were adjusted for age, sex, insurance status, diabetes, hypertension, CCT, IOP (only for CDR model), and CDR (only for IOP model). Statistically significant determinants of CDR included age, insurance, and IOP, while the only significant correlates of IOP were CDR and CCT. In terms of age, increasing age was predictive of larger CDR (*P* = 0.004). For each year increase in age, CDR increased by 0.002 (95% CI: 0.001–0.003). Patients with insurance also had smaller CDR than patients without insurance (*P* = 0.019). Compared to patients without insurance, patients with insurance, on average, had CDRs that were 0.062 smaller. As a determinant of CDR, for each mm Hg increase in IOP, CDR increased by 0.011 (95% CI: 0.008–0.013, *P*<0.001). For the model of IOP outcomes, CCT was the only determinant of IOP for which IOP increased by 0.022 mm Hg (95% CI: 0.011–0.032 mm Hg, *P*<0.001) per µm increase in CCT.

**Table 4:** Multivariate linear regression models for cup-to-disc ratio and intraocular pressure outcomes (n = 750).

	CDR^a^		IOP^b^	
Variable	β [95% CI]	*P*	β [95% CI]	*P*
Age	0.002 [0.001, 0.003]	0.004	−0.015 [−0.047, 0.017]	0.371
Sex (male)	0.025 [0.003, 0.053]	0.077	−0.569 [−1.306, 0.168]	0.130
Insured	−0.062 [−0.113, −0.010]	0.019	1.079 [−0.288, 2.446]	0.122
Diabetes	−0.034 [−0.075, 0.006]	0.095	0.974 [−0.095, 2.044]	0.074
Hypertension	0.023 [−0.008, 0.054]	0.143	0.345 [−0.476, 1.166]	0.410
IOP	0.011 [0.008, 0.013]	<0.001	N.A.	N.A.
CDR	N.A.	N.A.	7.630 [5.562, 9.697]	<0.001
CCT	0 [−0.001, 0.001]	0.168	0.022 [0.011, 0.032]	<0.001

CDR = cup-to-disc ratio; IOP = intraocular pressure; N.A. = not applicable; CCT = central corneal thickness.

a Model Performance: *R* ^2^ 0.33 with 578 degrees of freedom. Model was adjusted for age, sex, insurance status, diabetes, hypertension, CCT, and IOP.

b Model Performance: *R* ^2^ 0.34 with 578 degrees of freedom. Model was adjusted for age, sex, insurance status, diabetes, hypertension, CCT, and CDR.

## Discussion

No reported study has examined the extent of suspected glaucoma among Afro-Caribbean people residing in the U.S nor evaluated suspected glaucoma in individuals less than 40 years old in this population. Within the Haitian Afro-Caribbean population in South Florida, we found that 25.5% of 750 eligible individuals with no prior diagnosis of glaucoma or glaucoma suspect and who do not obtain regular eye exams were identified as glaucoma suspects. The results support the assumption that individuals of African descent have glaucomatous disease earlier in life and may explain why they have higher rates of glaucoma-associated blindness than those of non-African descent. Among individuals younger than 40 years old in our study population, 20.9% (CI 17.9%–23.9%) were identified as glaucoma suspects with both high IOP and CDR.

The population also had a mean IOP of 19.2±4.5 mm Hg, higher than that reported in the Barbados Eye Study (BES) of 18.0±4.1 mmHg [16], suggesting that Afro-Caribbeans, particularly those of Haitian descent, in the U.S. have even higher eye pressures than those outside the U.S. Elevated IOP was not the result of increased CCT because mean CCT among participants who met IOP criteria was comparable to mean CCT among all participants. Intraocular pressure was a more valuable means of defining glaucoma suspect status than was CDR, which is an important differentiation since healthy blacks in the U.S., and possibly Afro-Caribbeans, have larger CDRs [17]. A large CDR is not an accurate criterion in the evaluation of glaucoma in individuals of African descent and further emphasizes the importance of elevated IOP in this population.

In the BES, the estimated glaucoma suspect prevalence was 26.1% [11]. In the BES, glaucoma suspects were defined as individuals who met either VF criteria of two abnormal VF tests or optic nerve criteria. Optic nerve criteria were met if nerves showed at least two signs of optic nerve damage, including a CDR of 0.7 or greater. The BES prevalence of 26.1% was comparable to our result of 25.5%, despite the fact that we did not include VF criteria. Foster and colleagues reported that in glaucoma prevalence surveys, an accurate CDR threshold to identify glaucoma cases is 0.7 or greater [18]. Congruent with this finding and the criteria used in previous studies of glaucoma suspects among individuals of African descent, our study also employed a CDR of 0.7 or greater in addition to IOP criteria. Nonetheless, more individuals met IOP criteria than CDR criteria (16.8% and 14.1%, respectively) with a 5.5% overlap of individuals meeting both criteria.

A large, population-based study in St. Lucia also examined glaucoma in Afro-Caribbean people, and is the only other study to investigate this population outside the U.S. [10]. Mason and associates found that the prevalence of glaucoma was 8.8%. Mean IOP (17.7±4.3 mm Hg) was also lower compared to our study and the BES. Moreover, all three studies, including our own, showed that as age increased, the proportion of glaucoma suspects increased. In our study we adjusted for all covariates included in data collection to show that increasing age was a determinant of increased CDR (β = 0.002, *P* = 0.004). Despite this finding, the proportions of suspected glaucoma were high in both the youngest and oldest age groups (Table 3). This suggests that glaucomatous disease begins early in our study population and progresses with age.

The underlying reasons for the high rate of glaucoma suspects in this population are not clear and possibly genetic. In our study, 19.5% of all participants reported a family history of glaucoma, and 39.1% of these individuals stated a history of glaucoma in a first-degree relative. A *CDKN2B-AS* gene variant, identified initially in Caucasian populations [19], [20], is associated with primary open-angle glaucoma (POAG) in the Afro-Caribbean population of Barbados [21]. Nevertheless, the genes for POAG in individuals of African ethnic origin remain largely unknown. Performing a genome wide association study to discover the genetic biomarkers for POAG and glaucoma-related traits may provide more insight. The data suggests that environmental differences between South Florida and Barbados do not account for the high burden of glaucoma suspect status since results of IOP, CDR and CCT were very similar to the Barbados study. The high rate of suspected glaucoma in individuals less than 40 years old is also consistent with a genetic etiology. Patients in our study may have also been related to one another, elevating the rate of glaucoma suspects. Attempts were made to determine the degree of relatedness among individuals with a family history of glaucoma; however, retrospectively, we were unable to determine if patients were related to one another using the data initially collected.

The high prevalence of diabetes within this population could also serve as the reason for the high rate of suspected glaucoma. Cross-sectional studies have found a positive association between diabetes and POAG [22]–[24], while two prospective studies found significant positive associations between diabetes and incident POAG [25], [26]. In the BES, diabetes was also associated with an increase in IOP after four years’ follow-up [27]. Among our total study population, 14.5% had diabetes compared to the prevalence in non-Hispanic whites in the U.S. of 10.2% [28]. After adjusting for covariates, diabetes was not a statistically significant determinant of CDR or IOP; however, the negative association between diabetes and CDR and the positive association between diabetes and IOP trended towards significance (*P* = .095, *P* = .074, respectively). This could support the suggestion that diabetes may protect the optic nerve from elevated IOP [29]. In terms of elevated IOP, the BES prospectively demonstrated a positive association between IOP and the development of glaucoma [30]. Further studies are needed to demonstrate the association between this group’s elevated mean IOP and glaucoma.

Socioeconomic risk factors for suspected glaucoma within this population also exist. Immigration status serves as a formidable barrier to seeking medical care, including eye care. In 2000, the U.S. Citizenship and Immigration Services estimated that there were at least 76,000 undocumented Haitian immigrants in the United States, and the State of Florida has the highest number of these Haitian immigrants [31]. The patients included in this study had not received an eye exam within the last ten years, which may have been partially due to immigration status. In our analysis, being insured was a statistically significant determinant of CDR, indicating that patients with insurance had lower CDRs (−0.062, P = 0.019). Stein *et al.* investigated the relationship between insurance status and receiving glaucoma treatment in the US, and found no association between private insurance and receiving or not receiving treatment. However, Medicaid and Medicare beneficiaries were less likely to receive medical or surgical treatment for glaucoma [32].

Several limitations to our study may have overestimated the number of individuals with suspected glaucoma. The presence of selection bias cannot be ignored since this study was a retrospective study and patients may have attended the screening because of a vision problem. The VF test could have been replicated a third time if results were abnormal because in the non-cases group, there were a high proportion of abnormal tests (30.1%, CI 26.1%–34.2%) – implying that either non-cases have VF defects or participants did not understand the test. Cup-to-disc ratio was also measured subjectively with direct ophthalmoscopes on non-dilated eyes, potentially skewing the results. However, CDRs were comparable between those observed by direct ophthalmoscopy on non-dilated eyes and those re-examined by ONH photographs read by ophthalmologists (Pearson’s coefficient = 0.93 [*P*<0.001] for right eyes and 0.89 [*P*<0.001] for left eyes). Data could also have been collected on whether or not a patient had a family member participate in the study. We were unable to retrospectively determine if patients were related, which could elevate the rate.

To improve ophthalmic care, several efforts were made to provide counseling and follow-up, especially for patients with alarmingly high IOP. Patients documented as glaucoma suspects were given referrals with their test results for follow-up with their primary care providers or recommended ophthalmologists in the community. If participants did not have their own physicians or lacked health insurance, participants were referred to the local public hospital, which offers ophthalmic care to all county residents. Future eye examinations are planned to take place at the same community health center with the addition of experts on insurance plans to receive follow-up care, especially for younger patients with elevated IOP. The value of targeting younger Haitian Afro-Caribbean individuals in ongoing screening efforts is apparent based on the results of this study. Our personal observation from work we are doing in Haiti is in agreement with the conclusions of this study. Whether screening will influence the outcomes of glaucoma in this specific population remains to be determined in future studies.
